# Efficacy of krypton laser photodynamic therapy for oral mucosa dysplasia in 9,10-dimethyl-1,2-benzanthracene-treated hamsters

**DOI:** 10.3892/ol.2013.1554

**Published:** 2013-08-30

**Authors:** LINGYUE SHEN, QING XU, PINGPING LI, GUOYU ZHOU

**Affiliations:** Department of Oral and Maxillofacial Surgery, Ninth People’s Hospital, Shanghai Jiao Tong University School of Medicine, Shanghai, P.R. China

**Keywords:** photodynamic therapy, krypton laser, oral dysplasia, hamster

## Abstract

The present study aimed to evaluate the efficacy of krypton laser photodynamic therapy (PDT) with PsD-007 for the treatment of oral mucosa dysplasia in 9,10-dimethyl-1,2-benzanthracene (DMBA)-treated hamsters. A DMBA-induced hamster cheek pouch model of precancerous lesions was created and the resultant 25 hamsters were divided into five groups. The right side was treated with PDT and the left side was used as the positive control. Following systemic anesthesia, an incision was made in the groin area to expose the femoral vein. PsD-007 was administered intravenously through the femoral vein. Various doses of photosensitizer were used to treat groups A–E. Subsequent to closing the incision, the right side of the buccal mucosa was irradiated with light using the krypton laser at a wavelength of 413 nm, a power density of 150 mW/cm^2^ and an irradiation time of 20 min. At six weeks post-surgery, the response was analyzed using histological examinations of the buccal pouch mucosa. A total of 24 hamsters completed the six-week observation period, as one hamster from group C died in the second week following the PDT. Of all 24 irradiated sides, 15 formed normal mucosal tissues and nine demonstrated mild dysplasia. Of the total control sides, six developed moderate dysplasia, five developed severe dysplasia and 13 progressed to carcinoma *in situ* or squamous cell carcinoma (SCC). The results revealed a significant difference between the two sides (P<0.01) and the various doses of the PsD-007 groups. When the PsD-007 dose was >10 mg/kg, there was no statistical difference (P>0.05). PsD-007-mediated krypton laser PDT is effective for the treatment of oral mucosa dysplasia in hamsters.

## Introduction

Photodynamic therapy (PDT) is a new technology for the treatment of cancer that has gained interest within the past two decades and has been used in the treatment of lung, esophageal, cervical, bladder and skin cancer and other tumors (1). Clinically, PDT has been considered to be more applicable to tumors with a wide range of superficial locations, as it may be combined with fluorescence diagnostic techniques to distinguish the range of lesions, selectively kill tumors and reduce the damage to normal tissues. It is recognized that the efficacy of PDT in tumors with a depth of 1.0 cm is extremely high. Precancerous lesions of an oral or maxillofacial location are more superficial, therefore, PDT has a measurable effect on these types of lesions (2).

The present study used intravenous PDT to treat precancerous lesions in order to explore the mechanism of the treatment. Through the establishment of a golden hamster leukoplakia model, which was similar to human leukoplakia, the effect of the hematoporphyrin monomethyl ether (PsD-007)-mediated krypton laser PDT on the oral dysplasia of hamster was observed and the dose-effect correlation of PDT was also studied. This provides a basis and reference for the treatment of human precancerous lesions in the clinic.

## Materials and methods

### Experimental animals and establishment of leukoplakia model

#### Materials

A total of 25, six to eight-week-old female Syrian hamsters (n=25) were purchased from the BK company (Shanghai, China). 9,10-Dimethyl-1,2-benzanthracene (DMBA) was purchased from Sigma (St Louis, MO, USA) and the krypton ion laser was obtained from Coherent (Santa Clara, CA, USA). The hematoporphyrin monomethyl ether, PsD-007 (Shanghai Second Military Medical University, Shanghai, China), was prepared prior to performing the study.

#### Grouping

The 25 Syrian golden hamsters were randomly divided into five groups (A, B, C, D and E; n=5). The concentration of the photosensitizer used was 5, 10, 20, 40 and 60 mg/kg, respectively in the five groups. In all the animals, the cheek pouch of the right side was set as the PDT group and the left side was set as the control group.

#### Preparation of leukoplakia model

According to the methods used by Salley (3), 0.5% DMBA (5 g/l) was dissolved in acetone solution and stored in brown bottles. The cheek pouch was everted using tweezers and a number one oil painting pen dipped with 0.5% DMBA acetone solution was used to brush the tissues 10 times within a 1-cm range of the cheek pouch vein. The other side of the cheek pouch mucosa was prepared using the same method. The hamsters were made to fast for 2 h following the drug-smearing. All the animals were treated with the surface drug on Monday, Wednesday and Friday continuously for six weeks in the same manner. The study was approved by the ethics committee of Shanghai Ninth People’s Hospital Affiliated to Shanghai Jiao Tong University, School of Medicine (Shanghai, China).

### Krypton laser PDT

#### Femoral vein exposure

Six weeks after the drug-smearing, all the animals were made to fast for 12 h, were weighed and their skin prepared. Barbiturates (0.8–1 ml/kg; Changchun Medical Sciences, Changchun, China) were intramuscularly injected as a general anesthetic. The hamster was placed in a supine position and fixed to a special operating table. Using bromogeramine iodine tincture cotton balls to disinfect the skin of the groin, the hair was cut and the skin was disinfected again. A skin incision was made between the pubis and iliac crest to expose the femoral vein (>1 cm) and then the fascia was carefully peeled off for the venipuncture.

#### Femoral venipuncture

The femoral vein was pierced with a number 4.5 scalp needle. The photosensitizer, PsD-007, was injected slowly, and the breathing and heartbeat of the hamster were observed closely during the infusion process. Finally, the needle was removed and the incision was sutured layer by layer. Chlortetracycline eye ointment was coated on the wound.

#### Krypton laser irradiation

All the animals remained fixed to the operating table. The upper and lower incisors were wound with fine wire to keep the mouth open. The cheek pouch lesion was exposed and the krypton laser vertically irradiated the lesion at a wavelength of 413 nm, a power density of 150 mW/cm^2^ and an irradiation time of 20 min. The non-irradiated skin and mucous were shaded from the radiation using black stickers. Dexamethasone was administered to all the animals at three days post-surgery (0.1 ml, bid, im).

### Post-operative observation

#### Naked eye observation

The dynamic change in the experimental-side cheek pouch was observed and recorded.

#### Pathological observation

Six weeks after the surgery, all the animals were sacrificed and the cheek pouch mucosa specimens of the PDT and control groups were collected and fixed with 10% formalin for 48 h. The samples were conventionally dehydrated, embedded in paraffin and sliced. The slides were stained using hematoxylin and eosin (HE) and observed under a light microscope. The pathological diagnosis was concluded by two pathologists.

#### Observation indices

The pathological criteria, according to the 12 diagnostic criteria of epithelial dysplasia proposed by the WHO Oral Precancerous Lesions Collaborating Centre (4), were as follows: 2, mild dysplasia; 2–4, moderate dysplasia; and >5, severe dysplasia. The typical pathological changes of the tumor cells were recorded as carcinoma *in situ*.

The efficacy evaluation was based on the post-operative cheek pouch mucosal pathology, which was graded as a cure (normal mucosa) or as effective (mild dysplasia). The lesions were alleviated compared with the control side.

#### Statistics

Values are shown as the mean ± SEM. The differences between all the groups were evaluated using one-way ANOVA with a post-hoc Student-Newman-Keuls multiple comparisons test. The statistical analyses were performed using SPSS software (v13.0; SPSS, Inc., Chicago, IL, USA) and P<0.05 was considered to indicate a statistically significant difference.

## Results

### General observation

**Figure 1:**
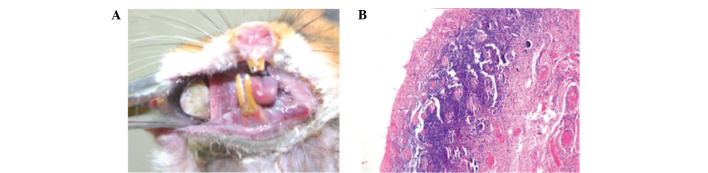
Observations following the treatment. (A) A necrotic tissue covering with white pseudomembrane on the buccal pouch mucosa was observed one to two weeks subsequent to PDT. (B) One week after PDT (HE staining; magnification, ×40). Shedding of the epithelial layer, ulcer formation and inflammatory cell infiltration in the lamina propri and muscularis is observed. PDT, photodynamic therapy; HE, hematoxylin and eosin.

In groups A–E, the laser-irradiated cheek pouch mucosa presented with mild edema immediately after the PDT and with reactive edema involving the tongue and neck within 48–72 h. The swelling subsided after five to seven days. One week after the surgery, the cheek pouch mucosa became necrotic and grayish yellow. The pouch formed an ulcer and the necrotic tissues formed a white pseudomembrane to cover the mucosa within one to two weeks (Fig. 1). Subsequent to approximately four weeks, the wound healed. However, certain hamsters demonstrated slight scarring.

### Effect of PDT on dysplasia

**Figure 2:**
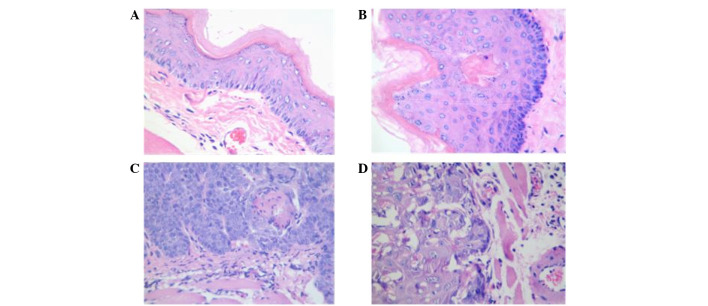
HE results of PDT after six weeks. (A) The PDT-treated side of the cheek pouch mucosa in group B (HE staining; magnification, ×40). (B) The PDT-treated side of the cheek pouch mucosa in group C. Mild dysplasia and dyskeratosis (arrow) were observed (HE staining; magnification, ×40). (C) Control side of the cheek pouch mucosa in group C. Severe dysplasia was observed. (HE staining; magnification, ×40); (D) Control side of the cheek pouch mucosa in group E (HE staining; magnification, ×40). PDT, photodynamic therapy; HE, hematoxylin and eosin.

Of the total 25 hamsters, only one hamster from group C died. Among the remaining 24 hamsters in the PDT group, 15 cases exhibited normal mucosa and nine displayed mild dysplasia. In the control group, six cases presented with moderate dysplasia, six with severe dysplasia and 13 with carcinoma *in situ* and SCC (Table I; Fig. 2). There was a significant difference between the PDT group and the control group, as shown by the χ^2^ test (P<0.01).

### Correlation between dose and effect of PsD-007

As shown in Table II, the cure rate in group A was 20%. There was a significant difference between group A and the other groups (P<0.05). The cure rate in groups B, C, D and E was 80, 75, 80 and 60%, respectively, indicating that the cure rate did not increase with increasing concentration (P>0.05) after a concentration of 10 mg/kg. Therefore, 10 mg/kg was defined as the effective dose for the treatment of dysplasia using krypton laser PDT.

### Side-effects

A single hamster in group C (20 mg/kg) died of a photosensitive reaction two weeks after the surgery. In the other groups, the hamsters presented various degrees of photosensitive reactions, particularly in groups D (40 mg/kg) and E (60 mg/kg). There were three cases in group D and four cases in group E that demonstrated photosensitive reactions, including a rash, alopecia and scab formation.

## Discussion

The precancerous lesions of the cheek pouch hamster model are considered the best choice for observing the dynamic process from precancerous lesions to cancer, which may be used to observe and evaluate the effect of PDT and obtain reliable results. The golden hamster used in the present study is a small mouse and the tail is extremely short. Therefore, a subcutaneous superficial vein or tail vein injection would be unsuccessful. Consequently, the photodynamic methods of the present study were established using a femoral vein injection, which has repeatability as a new method.

The mechanism of the destruction of the tumor using PDT may be summarized as follows: i) The hematoporphyrin derivative photosensitizer is combined with a biofilm system, particularly the cell plasma and nuclear membranes, to destroy the mitochondria, lysosomes and liposomes of the tumor cells. Therefore, the activities of the cells that are necessary for life, including respiration, electron transport and ATP synthesis, are affected due to the mitochondrial damage, thereby inducing cell apoptosis (5–7); ii) PDT causes tumor microvascular dysfunction and structural damage, including destruction of the vascular endothelium, platelet adhesion, degranulation, siltation of the blood cells and vascular occlusion, thereby leading to tumor necrosis (8); iii) PDT may cause an immune response of the tumor cells responding to tumor antigens (9); and iv) PDT may affect the gene expression of the tumor cells (10). However, due to the shorter photosensitizer absorption lines, the penetration depth of the corresponding laser is limited. Therefore, deep and large tumors may not be able to be treated using this method. It has been recognized that the method is only efficient on tumors with a depth of ≤1.0 cm (11,12).

Precancerous lesions are extremely superficial and only identified in the epithelium, which means that the desired treatment results may be obtained with PDT. PDT has gradually been focused on the treatment of superficial tumors and precancerous lesions. The treatment of precancerous lesions with PDT and 5-AminoLevulinic Acid (ALA) has become a focus for research (3). ALA is an exogenous photosensitizer, which is able to indirectly synthesize protoporphyrin IX at a 635 nm absorption peak *in vivo* following absorption through the skin or mucous (14). ALA-PDT has been used to treat a variety of superficial precancerous lesions, including skin, bladder, lung and gastrointestinal cancer. Tsai *et al*(15) revealed that a combination of 20% ALA and a light-emitting diode (LED) laser (630 nm, 100 J/cm^2^) was able to effectively treat hamsters with oral dysplasia induced by DMBA. Furthermore the same ALA-PDT method was used to treat 32 patients with oral dysplasia. Following a six-month follow-up period, the results revealed that a complete response was evident in seven cases and a partial response was evident in 13 cases. the treatment was ineffective in 12 cases. Kübler *et al*(16) used 20% ALA to treat 12 patients with leukoplakia. After 2 h, the patients were irradiated using a 630 nm laser (100 J/cm^2^). The results revealed that a complete response was evident in five cases and a partial response was evident in four cases. The treatment was ineffective in three cases. The lesions disappeared following re-treatment in one case.

The systemic toxicity was observed to be low when partially coated with ALA, and light did not have to be avoided following the surgery due to a rapid metabolism (15,16). However, the efficacy of ALA-PDT was dependent on the level of protoporphyrin that was synthesized in the tumors. The capacity of synthesizing protoporphyrin has yet to be elucidated in various tumor cells and precancerous lesions. Therefore, the method lacks a theoretical basis for the treatment of precancerous lesions. In the present study, the intravenous PDT method was used, in which the mechanism has been confirmed. PsD-007 was the purified product of the hematoporphyrin derivatives and the maximum absorption peak was 408 nm, which was close to the wavelength of the krypton laser (413 nm). The results revealed that the overall cure rate of dysplasia was 62.5% (15/24) in the PDT group, indicating that PDT had a positive effect on the precancerous lesions.

The traditional view is that the porphyrin-based photosensitizer has a selective storage role for tumor tissues. However, there has not been sufficient experimental evidence for this. The mechanism behind the treatment of tumors using PDT was believed to function as the new tumor tissues were rich in blood vessels. The hematoporphyrin derivative photosensitizer reached a relatively high concentration in the tumor tissues through the blood vessels, thereby selectively destroying the tumors. The objective of the present study was to treat oral precancerous lesions, in which the vascular distribution is not clear. Therefore, a confirmation of the differences between photosensitizer concentrations in the precancerous lesions and normal tissues was required. In the present study, all the animals demonstrated mucosal necrosis and shedding following the treatment with PDT. The selectivity was also determined by the range of the irradiation. However, the normal tissues were also damaged, which may have been due to fact that the vascular distribution of the precancerous lesions was not abnormal compared with the tumors. Therefore, the difference in the concentration of the photosensitizer was not great enough between the precancerous lesions and the normal tissues in order to obtain selective damage results.

The results of the present study revealed that 15 cases were cured in the PDT group at six weeks post-treatment, none of which appeared carcinogenic. However, in the control group, 13 cases appeared carcinogenic, indicating that the efficacy was significantly improved in the PDT group. Furthermore, although the animals demonstrated mild dysplasia in the PDT group, the degree of dysplasia was less than that of the control group. This provides evidence for the efficiency of PDT using a krypton laser and PsD-007 on the precancerous lesions of the golden hamsters. In addition, there was a significant difference between group A (5 mg/kg) and the other four groups (>10 mg/kg). However, although the cure rate did not increase with an increasing concentration, the phototoxicity did. Therefore, 10 mg/kg was considered a suitable photosensitizer concentration. A single hamster in group C died of a severe swelling response and photosensitive reaction at two weeks post-surgery. In groups D and E, the animals also demonstrated photosensitive reactions, including a rash, alopecia and skin ulceration. These reactions may have been due to the larger dose of photosensitizer and the unintentional exposure to light.

Although PDT exhibited a positive effect on the precancerous lesions, the lesions had characteristics of regional malignancy and multi-step carcinogenesis. Therefore, the efficiency of the treatment may have been reduced and the lesions may have recurred after an extended observation time. PDT requires further study in order to evaluate the long-term effects of the method on precancerous lesions.

## Figures and Tables

**Table I:** Pathological results of the PDT-treated and control sides of the buccal pouch mucosa in groups A–E following six weeks of treatment.

			Epithelium dysplasia, n (%)			
				
Group	n	Normal mucosa, n (%)	Mild	Moderate	Severe	Carcinoma *in situ*, n (%)
A						
PDT-treated side	5	1 (20.0)	4 (80.0)	0 (0.0)	0 (0.0)	0 (0.0)
Control side	5	0 (0.0)	0 (0.0)	1 (25.0)	1 (25.0)	3 (60.0)
B						
PDT-treated side	5	4 (40.0)	1 (60.0)	0 (0.0)	0 (0.0)	0 (0.0)
Control side	5	0 (0.0)	0 (0.0)	2 (40.0)	1 (20.0)	2 (40.0)
C						
PDT-treated side	4	3 (75.0)	1 (25.0)	0 (0.0)	0 (0.0)	0 (0.0)
Control side	4	0 (0.0)	0 (0.0)	0 (0.0)	1 (25.0)	3 (75.0)
D						
PDT-treated side	5	3 (60.0)	2 (40.0)	0 (0.0)	0 (0.0)	0 (0.0)
Control side	5	0 (0.0)	0 (0.0)	2 (40.0)	1 (20.0)	2 (40.0)
E						
PDT-treated side	5	4 (80.0)	1 (20.0)	0 (0.0)	0 (0.0)	0 (0.0)
Control side	5	0 (0.0)	0 (0.0)	1 (20.0)	1 (20.0)	3 (60.0)
Total						
PDT-treated side	24	15 (62.5)	9 (37.5)	0 (0.0)	0 (0.0)	0 (0.0)
Control side	24	0 (0.0)	0 (0.0)	6 (25.0)	5 (20.8)	13 (54.2)

PDT, photodynamic therapy.

**Table II:** Cure and effective rates of groups A–E.

Group	Dose, mg/kg	Cure, n (%)	Effective, n(%)	n
A	5	1 (20.0)	4 (80.0)	5
B	10	4 (80.0)	1 (20.0)	5
C	20	3 (75.0)	1 (25.0)	4
D	40	3 (60.0)	2 (40.0)	5
E	60	4 (80.0)	1 (20.0)	5
Total	-	15 (62.5)	9 (37.5)	24
